# Up-To-Date Kitchen Specialties

**Published:** 1912-04-27

**Authors:** 


### Up-to-date Kitchen Specialties.

### THE "VICTORIA" VEGETABLE-PARING MACHINE.

The question of the paring of vegetables has always been a serious item in the administration of the institution kitchen, and until comparatively recently the only type of apparatus available for the performance of this work in quantities has been the "nutmeg-grater" variety of potato-peeling machine. In a large number of institutions this form has been tried and found wanting on account of the unavoidable mess and waste, and also the rapid wear of the apparatus. During the last few years an efficient and satisfactory machine has been placed on the market; one which will peel not only potatoes, but also carrots, turnips, parsnips, and even swedes. This is the "Victoria" vegetable-paring machine, manufactured by the Imperial Machine Co., of 11 Crown Parade, Cricklewood, N.W. In this machine the perforated metal sheet of the old nutmeg-grater type is replaced by a lining of granular carborundum, which is fused on to the metal. The life of the apparatus treated by this method is such that some of the machines in actual operation to-day have already peeled over 1,500 tons of vegetables without any repairs. The waste with these machines is very small in amount, and the mess in the kitchen is entirely obviated. Anyone interested in the rapid peeling of vegetables cannot do better than investigate this system, which is now in use in a number of institutions.

### THE "RAPID" POTATO-PEELING MACHINE.

Although very many kitchen appliances have been invented or provided by Mabbott and Co., Poland Street, Manchester, their apparatus of chief interest to administrators of institutional and hospital kitchens is the potato-peeling machine. The firm has been perfecting the type of this labour-saving device for many years, for it has taken considerable time and trouble to devise an apparatus which shall combine smallness of size, cleanliness of working, and speed of output. Willanks No. 3 "Rapid" potato-peeling machine is the result of much labour and human ingenuity. It is of particular interest to institutions, for it does not involve great capital outlay which, for hospital workers at any rate, is of the first importance. It costs £3, and will work and peel 300 lb. of potatoes per hour. Two larger machines are also obtainable, which will deal respectively with 15 lb. and 30 lb. per minute, No. 3 and No. 4 "Premier." Finally, all the machines of this firm are fitted with ball-bearings, a point of the utmost importance to those who constantly in the past have had to meet the complaints of the staff that the machine is hard to work. Those designed for hand-power are made for a girl to turn. Illustrations of the peeler will be found in our advertisement columns.

### WELBANK'S BOILERETTE.

The large collection of appreciative opinions on the reliability of this excellent cooking apparatus already in possession of the manufacturers leave but little to say about it. We need scarcely do more than to draw our readers' attention to this ideal saucepan, and to reiterate the favourable opinion expressed upon it in a former issue. For sick-room cookery the Welbank boilerette is invaluable; but there are hardly any circumstances under which it will not be found more effective and convenient than other methods. The inner receptacle, in which the food is placed, is encased by a steam jacket. The safety-valve obviates all necessity for attention whilst the food is boiling, for neither burning nor boiling-over can occur. Doctors and nurses not only can recommend this saucepan with perfect safety, but will confer a boon on those to whom it is introduced. It is one of the few articles which recommends itself when once it has been tried. The makers are R. W. Welbank and Co., of Banbury, and 105 Newgate Street, E.C.

### A NOTEWORTHY CATALOGUE.

The reputation of the firm of James Slater and Co. is a high one, and it is easy to see that it has been built upon a solid foundation. The apparatus of this firm stand in a class by themselves; their machines have stood severe tests in institutions all over the country, and the firm claim that they are examples of the highest quality in material and workmanship. Such qualities cannot be produced at competitive prices, but it is a false economy to save a hundred pounds in the first cost of a large installation, if an annual loss is incurred by extra fuel and frequent breakdowns. We have no hesitation, therefore, in strongly recommending institution authorities to consider carefully the catalogue issued by Slater and Co., of Wells Street, W., before embarking on the purchase of any sort of cooking apparatus, warming or hot-air service plant. Hospitals will find that their needs, apart from the kitchen, are also provided for. In particular, we can speak from practical experience of the steam instrument sterilisers supplied by Slaters. They possess a convenience and efficiency combined with a durability that we have proved cannot easily be surpassed.

### THE "CORNHILL" POTATO-PEELING MACHINE.

The Avamore Engineering Company, Ltd., of Kingsway House, London, has set itself the problem of avoiding the waste that attends all hand forms, and some machine methods, of peeling potatoes. The result is the machine above mentioned, which is guaranteed for two years, and is designed to save 15 per cent. on every potato over that peeled by hand. It is made in two sizes, which will peel 15 lb. and 40 lb. respectively in less than one minute. The machine has a very simple action, which alone is enough to recommend it to all hospital administrators. The price of the "Cornhill," Nos. 1 to 3, is £26 10s., and any of our readers in want of a new machine of this kind should certainly consult the firm for details and price lists.

### HOW TO DEAL WITH CUTLERY.

The risk of most contrivances for cleaning knives and cutlery is that they shorten the lives of the knives which they clean. The latest developments, therefore, in this direction have sought to avoid this defect, and the "Acme Cutlery Renovator," made by the Avamore Engineering Company, of Kingsway, London, is so far protected as to clean and polish the entire blade in one operation, without straining or bending the handles, or destroying the blades. Its capacity ranges from 800 to 1,500 knives per hour, and a price list will be sent to all inquirers.

### AN ICELESS REFRIGERATOR.

With last summer's heat apparently returning in early time this year, and the lesson of the railway strike that ice may always become difficult to procure, an iceless refrigerator at once claims attention. The "Arktos," made by the Avamore Engineering Co., which has offices in Kingsway, London, has much to recommend it. It requires no ice or motive-power, being operated by gas or oil burners, or by a steam coil. It is made in all sizes and types, and should be carefully studied by every administrator who is anxious to remedy the inconveniences of an ice-plant. A card to the firm, mentioning The Hospital, will bring all particulars at once. It need be added here only that the "Arktos" is operated on the ammonia-absorption principle, and its essential parts are a combined absorber and generator or still, a condenser, and a receiver. Ice-making, dairy cooling, freezing meat, drying air, cooling rooms and stores are a few examples of its multifarious adaptiveness.

### A NEW LABOUR-SAVING APPLIANCE.

The modern institutional kitchen has become as scientific in its methods as any other department, and the value of a kitchen and its administration can largely be measured by the labour-saving appliances which it contains. We therefore have no hesitation in drawing attention to the "Vortex" dish-washing machine, which is manufactured by the Avamore Engineering Co., Kingsway, London. This machine washes, cleans, dries, polishes, and even sterilises dishes; having no gears it can claim to be noiseless, and the risk of breakage is practically nil. The latest model has square tanks and baskets, a great improvement on the round pattern. Arrangements can be made for inquirers to see the "Vortex" actually working at many of the institutions in which it has been adopted. The "Vortex" is very highly spoken of, and though its interesting details of construction would be out of place here, any reader who will write to the firm, mentioning The Hospital, will receive every particular of price and specification.

### THE GAS FIRE AND GAS COOKERY.

At the Exhibition and Annual Nursing and Midwifery Conference at the Horticultural Hall this week the Gas Light and Coke Company's exhibit suggests a room adjoining a medical ward, where coal fuel has been abolished, and where, consequently, there is no dust. The gas-cooker, fixed in a well-ventilated recess, provides a ready means of cooking dainty foods for invalid patients, the gas-cooker rendering the process very clean. The well-ventilated gas fire is fitted to warm the room. Such fires are invaluable in a bedroom, where evenness of temperature and absence of noise from poking coal fires are required. Another point is that by installing a gas fire a down-draught in a chimney can be made impossible. Then hot water is obtained from the gas circulator, which takes the place of the kitchen coal-range boiler, and renders hot water available at any hour of the day or night. The circulator is fitted with a thermostat; when the water has attained the required temperature the gas supply is automatically cut off, and automatically lights up as the temperature of the water is reduced. The gas steam-radiator is fitted with a rail for drying towels. The gas distilling apparatus will distil water in quantities of about three pints per hour. There is also gas apparatus for the sterilising of surgical instruments. This is enough to show the manifold purposes to which gas can be applied. Its own cleanliness and convenience recommend it so strongly that such a list of its achievements is the best and most practical testimony to its value, and one which all hospital authorities will not fail to note.

